# Comparing Gas and Electrical Stunning: Effects on Meat Quality of Pigs When Pre-Stunning Physical Activity Is Minimal

**DOI:** 10.3390/foods10020319

**Published:** 2021-02-03

**Authors:** E. M. Claudia Terlouw, Véronique Deiss, Thierry Astruc

**Affiliations:** 1 INRAE, VetAgro Sup, UMR Herbivores, Université Clermont Auvergne, F-63122 Saint-Genès-Champanelle, France; veronique.deiss@inrae.fr; 2 INRAE, QuaPA, F-63122 Saint-Genès-Champanelle, France; thierry.astruc@inrae.fr

**Keywords:** pigs, gas stunning, electrical stunning, meat quality, stress, muscle pH, meat color

## Abstract

A total of thirty pigs were experimentally slaughtered using gas (80% CO_2_ in air, 90 s; 30% CO_2_/70% N_2_O; 90 s) or electrical stunning (1.3 A, 10 s). Stunning may accelerate post-mortem muscle metabolism, due to psychological stress and/or muscle contractions. The specific effects of the stunning method were studied by limiting pre-stunning physical activity and stress: pigs were driven in a trolley from the rearing to the stunning site (6.5 m) and immediately slaughtered. Bleeding efficiency and carcass characteristics were similar and satisfactory for all stunning methods. Early post-mortem pH decline in the *Longissimus lumborum* was faster following gas compared to electrical stunning. The pH of other muscles was not influenced; color and drip loss showed minor effects. Hence, results are in contrast to current beliefs: compared to electrical stunning, following gas stunning, the stress and muscle contractions during the induction of unconsciousness have a slightly greater impact on *Longissimus lumborum* muscle metabolism; differences are minor and limited to certain muscles only.

## 1. Introduction

At slaughter, before bleeding, pigs are stunned either electrically, or with high (>80%) CO_2_ concentrations. For electrical stunning, two electrodes are placed on either side of the head of the animal and a current of predetermined intensity crosses the brain. This causes a generalized epileptiform seizure; that is, a massive and synchronised depolarisation of the neurons, resulting in a brief period with greatly diminished brain activity causing unconsciousness [1]. For gas stunning, in EU countries, pigs are introduced into a gondola that is lowered into a 7 to 8 m deep pit containing at least 80% of CO_2_ at the bottom of the pit (Council Regulation (EC) No 1099/2009). Following inhalation, the CO_2_ causes acidification of the blood and subsequently, the cerebrospinal fluid [2]. Consequently, the neurons of the brain are acidified and are unable to function correctly, resulting in unconsciousness [3].

Various studies have compared the two techniques, relative to animal welfare issues and to meat quality. Most modern gas stunning systems allow pigs to stay in groups during the stunning process, considered an advantage for animal welfare, as pigs are gregarious. On the negative side, at a concentration of 80% CO_2_, the induction of consciousness needs 21 to 30 s, a period during which pigs exhibit apparent respiratory distress, as well as muscular contractions and convulsions [4,5,6,7,8,9]. Pigs may further express avoidance reactions, also indicative of the aversiveness of high CO_2_ concentrations [10,11,12]. The advantage of electrical stunning is its instantaneous induction of unconsciousness. However, automatic systems, which are mostly used in commercial settings, are equipped with a restrainer, which is stressful for the animals [13]. In addition, the automatic positioning of the electrodes makes it sometimes difficult to ensure that the intensity of the current passing through the brain of the animal is sufficient [14]. 

Generally, compared to electrical stunning, gas stunning is described as producing better meat quality. Earlier studies found that electrical stunning resulted in meat with a faster initial post-mortem pH decline, a lighter color, and increased drip loss associated with a higher incidence of Pale Soft Exudative like (PSE-like) meat [15,16,17,18,19]. Electrical stunning was further associated with higher incidences of petechiae and ecchymosis, especially in carriers of the n-allele (halothane sensitivity) [15,16,18,20]. These studies were, however, conducted under commercial or near-commercial conditions, that is, animals were walked to the stunning site. It is, therefore, unknown if the differences in meat quality express more pronounced effects of electrical compared to gas stunning on post-mortem muscle metabolism or whether the electrically stunned pigs were subjected to greater levels of physical activity and/or stress before stunning, which may also influence post-mortem pH decline [21].

The stun method itself may influence post-mortem muscle metabolism for various reasons. After a successful electrical stun, pigs exhibit a generalized epileptic seizure, first causing muscle contraction (tonic phase), followed by a series of alternating, sometimes violent, contractions and relaxations of the muscles (clonic phase). The seizure induces further hyperactivity of the peripheral nervous system and hypersecretion of hormones, including adrenaline [22,23]. The muscle contractions and release of hormones are likely to accelerate early post-mortem energy metabolism leading to a faster acceleration of post-mortem pH decline amongst others [21]. CO_2_ stunning causes stress during the induction period and provokes involuntary muscle contractions, all of which may also cause acceleration of muscle metabolism. Furthermore, CO_2_ inhalation causes the acidification of the blood [23], which could cause additional strain on the buffer system of muscle cells, reducing their capacity to maintain intracellular pH.

The objective of the present study is to investigate whether differences in pre-mortem activity levels contribute to the effects of different stunning methods, gas and electrical, on meat quality traits. The study took place under experimental conditions. To limit pre-mortem physical activity, pigs were conducted from their rearing pen to the stunning site in the adjacent slaughter room using a trolley. Electrical stunning was induced manually without use of a V-restrainer. For gas stunning, the two best performing gas mixtures of a precedent behavioral study [7], 80% CO_2_/air and 70% N_2_O/30% CO_2_, were used, immerging the pig and the trolley in the mixture as herein described. 

## 2. Materials and Methods

All aspects of the protocol were in accordance with the French legislation relative to animal protection in the context of experimentation, and all necessary official licenses for animal experimentation were up-to-date. The experimental rooms and equipment were the same as in a precedent study [7].

### 2.1. Animals and Housing

The study used 30 female pigs (Duroc × (Landrace × Large White)) weighing between 80 and 90 kg. Pigs were bought from a local producer (GAEC Petiot, Liernolles, France) 10 days before the trial and housed in an experimental breeding building located at the INRAE research center. They were kept in groups of 10 pigs in pens (3 × 4.5 m) with straw bedding in a single room. Each pig received 2.5 kg of standard concentrate per day and water was permanently available. Artificial light was on from 8 to 20 h. The experiment was organized in 3 consecutive series balanced for the experimental stunning procedures. The week preceding the trial, the two persons who would manipulate the pigs during the trial entered each pen daily and touched the pigs to habituate them to their presence.

### 2.2. Gas Equipment

**Figure 1:**
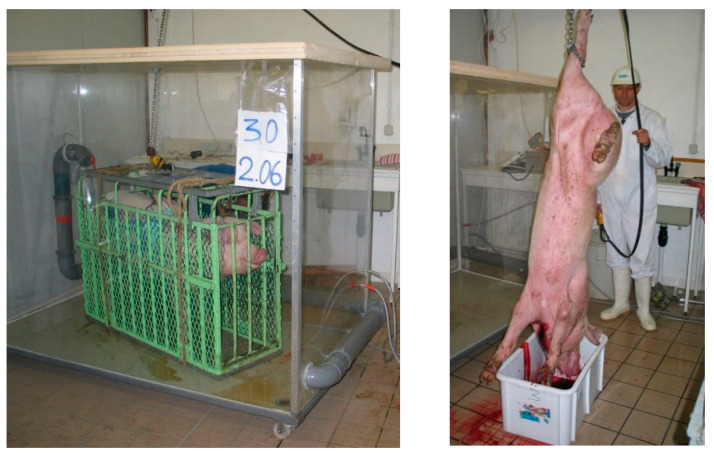
Experimental set-up: the gas chamber containing a pig (**left**) and the blood collection of the slaughtered pig (**right**).

Stunning and slaughter were carried out in the room adjacent to the rearing room described in our previous publication [7]. The room contained a 1 × 2 × 1.50 m gas chamber made from 1 cm thick Perspex glass and a PVC floor and mounted on wheels (Figure 1). The lid made from extruded polystyrene consisted of two halves to allow rapid closing and opening of the chamber. Grooves in the lid, both at the level where the lid joined the Perspex glass and where the two halves joined, allowed a relatively airtight closing of the chamber. Each half lid was equipped with a half PVC tube, vertically positioned, that joined each other in the middle to serve as a guide for the chain of the pulley that carried the cage with the pig. This tube was closed airtight between tests if the box contained gas. At a distance of 30 cm above the floor of the chamber, 10 cm from an angle, two connecting sleeves were permanently fitted on, to which gas tubes were attached to fill the chamber with gas. A closed circuit made from 10 cm PVC tubes and equipped with a ventilator (Systemair, Skinnskatteberg, Sweden) turning at a slow speed was fitted externally to the chamber to ensure homogeneous gas mixtures. It drew the gas mixture from the chamber in a corner 15 cm above the floor and returned it to the chamber in the diagonally opposite corner, at 97 cm above the floor. A sensor connected to a CO_2_/O_2_ analyser (Oxybaby gas analyser, Witt, Morsang sur Orge, France) was attached to the tube, at the level of the opening which the gas mixture was returned. The room was further equipped with manual stunning tongs, a slaughterline and a cold chamber kept at 4 °C.

The chamber was filled with the correct gas mixture before each slaughter session. To fill the chamber with the N_2_O/CO_2_ mixture, it was first filled with 100% of N_2_O, and subsequently, CO_2_ was added until it reached 30% while releasing the N_2_O outside the building.

### 2.3. Procedures 

Pigs were either gas (90 s exposure to 80% of CO_2_ in air, *n* = 10; or 70% of N_2_O with 30% of CO_2_, *n* = 10) or electrically stunned (manual Morphée tongs (Lelong & Cie, Savigny Le Temple, France), 230 V applied for 10 s, *n* = 10; Table 1). The system was not equipped to measure current intensity but was set to be around 1.3 A [24].

The day before slaughter, five pigs were separated from their rearing group and introduced in an adjacent 1.5 × 3 m pen without food but with access to water. The next day, a few minutes before slaughter, a pig was removed from its pen and introduced into an adjacent waiting pen (1.5 × 1.0 m) with vision on the pen mates. After 10 min, the pig was introduced into a cage (122 × 44 × 88 cm) built with metal bars, mounted on wheels and containing two vertically sliding doors and a removable floor. The pig was transported in the trolley over 6.5 m to the experimental room.

In the case of gas stunning, the chain of the pulley was attached to a hook in the middle of the top of the cage and lifted at a height of 2 m, attached to a slaughter line rail, moved above the chamber containing the correct gas mixture and descended onto the chamber floor (see Figure 1 in [7]). Duration between the moment of attachment and positioning on the chamber floor was 57 s [7]. The chamber was only briefly opened, to allow the introduction of the pig. Following the appropriate exposure duration, the cage with the pig was lifted again and descended on the floor of the experimental room. The bottom of the cage was detached, and the top of the cage was removed to have access to the unconscious pig. Recumbent position, and the absence of corneal reflex and breathing were verified to ascertain unconsciousness [25]. The pig was immediately shackled, hoisted and bled (stun-stick interval of around 40 s) by a trained slaughterman using the normal slaughtering procedure. Each pig was exposed for 95 s to one of the gas mixtures. As the descent and the lifting procedure comprised each time 15 s inside the box, the pigs remained for 75 s on the bottom of the chamber.

In the case of electrical stunning, the pigs were stunned immediately following arrival in the room through a large opening on the top of the cage, then the bottom of the cage was detached and the top removed. Recumbent position and the presence of generalized tonic muscle contraction were indicators of unconsciousness of the pig immediately after the stun; absence of corneal reflex and breathing were following hoisting and during bleeding [25]. The pig was shackled, hoisted and stuck before the end of the tonic phase (stun–stick interval of 10 to 15 s). The carcass was introduced in the scalding tank (Banss, 65 °C) following the end of bleeding. The remaining hairs were burnt using a gas burner and scraped with a knife to obtain a clean carcass before evisceration and splitting. The carcass was entered in the cold room 45 min post-mortem.

Each day, 5 pigs were slaughtered, on 3 consecutive days, during 2 weeks. Each day a single gas mixture was used on 3 or 4 pigs, and 1 or 2 pigs were electrically stunned. Gas mixtures were alternated over days. For each stunning method, 10 pigs were used, selected from different rearing groups in a balanced design. 

### 2.4. Measurements

Bleeding efficiency. The blood was collected over different intervals: 0–30 s, 30–60 s, 1–2, 2–3 and 3–5 min in five 50 × 30 × 20 cm recipients following the bleeding cut and the contents were weighed (Table 1).

Carcass weight. 30 h post-mortem, the head and the left half of the carcass were weighed. Carcass weight was estimated as the weight of the head and twice the weight of the half-carcass.

Muscle pH decline, temperature, glycogen and lactate contents. At different moments following slaughter, different variables related to muscle energy metabolism were evaluated in 4 different muscles: *Longissimus lumborum* (LL), *Semispinalis capitis* (SC), *Adductor femoris* (AF) and *Semimembranosus* (SM) (Table 2). Five minutes after bleeding, several grams of the LL muscle at the level of the sixth rib were excised to obtain 2 samples of 2 g and 1 g to determine pH, and at a later stage, glycogen/lactate content, respectively. To determine the pH, samples were immediately homogenised (Polytron, Steinhofhalde, Switzerland) during 20 s in 18 mL of 5 mM iodoacetate, and the pH of the homogenate was measured with a glass electrode (Inlab 427, Mettler Toledo, Greifensee, Switzerland), connected to a portable pH meter (Schött-Geräte, Germany). The samples for glycogen and lactate determination were immediately frozen in liquid N_2_ and stored at −80 °C until assaying.

At 30 and 60 min post-mortem, samples were excised from the LL, SC, AF and SM to determine pH as described above. At 30 h post-mortem, pH was measured directly on the carcass using a pH meter (WTW 340-B, Weilheim, Germany) equipped with a probe (Sentix SP, WTW, Weilheim, Germany). Temperature of the LL muscle was measured 5, 30 and 60 min and 30 h post-mortem, and of the SC, AF and SM muscles 30 and 60 min and 30 h post-mortem, directly on the carcass using an electronic thermometer equipped with a probe (TFK 150/E Weilheim, Germany).

Fractures and petechiae. The slaughterman verified the possible presence of bone fractures during splitting of the carcass and one of the experimenters was assigned to verify the presence of petechiae on a slice of the LL and SM muscles of similar size 30 h port-mortem.

Drip loss (DL). A total of thirty hours post-mortem, two slices about 1 cm thick were excised of each of the LL and SM muscles, weighed and suspended in a plastic bag and maintained at 4 °C. One (DL1), 2 (DL2) and 5 days later (DL5), after removing the water on the surface, each slice was weighed and suspended again on days 1 and 2). For the different time points, water loss was calculated as the percentage of the preceding weight [12]. Total DL refers to the weight loss on day 5 relative to the initial weight of the slice.

Rendement Napole (RN). This measurement is a standardized laboratory method for estimating yield of cured and cooked ham [26]. A total of thirty hours post-mortem, 100 g of the LL and SM muscles were excised, ground and mixed with 20 g of brine (136 g of nitrate salt/l water) in glass beakers. After closing them with a lid, the beaker remained at 4 °C for 24 h, and was subsequently placed in a water bath. The water bath was heated up to boiling temperature and maintained boiling for 10 min. The beakers were then removed from the water and the cooked meat placed on a draining rack at room temperature for 2.5 h before weighing. The Rendement Napole was calculated as the final weight relative to the initial weight expressed as a percentage.

Meat color. The color coordinates L*, a* and b* were measured 30 h post-mortem using a Minolta chromameter (CR-300, Minolta Corp. Osaka, Japan, no protective glass) equipped with a 0° viewing angle and using illuminant C on the surfaces of the LL, SM, and AF, after 1 h of blooming. Before measurement, humidity was gently removed from the surface using a clean tissue. For the SM color measurements, in order to standardize, the more internal, whiter part of the muscle was chosen [13].

### 2.5. Glycogen and Lactate Assays

Approximately 200 mg of lyophilized LL was ground and suspended in 10 mL of 0.5 M perchloric acid for 15 s with a homogenating device (Polytron, Steinhofhalde, Switzerland). Lactate and glycogen content were determined spectrophotometrically as described in an earlier paper [21], following methodologies described by [27,28]. Glycolytic potential (GP), the sum of compounds likely to produce lactic acid post-mortem, was calculated using the formula proposed by [29], to estimate muscle glycogen reserves at the moment of slaughter. In the calculation glucose and glucose-6-phosphate were excluded as they had not been determined but they have a minor contribution (between 4.5 and 7.5%; [29] to the GP. Values are expressed as µmol lactate equivalents per g of fresh tissue. GP of the LL and SM were based on samples collected 5 and 30 min post-mortem, respectively.

### 2.6. Statistical Analysis

XLStat (version 2018.1.1; Addinsoft 2020; XLSTAT statistical and data analysis solution, Paris, France) was used for comparisons between stunning methods and muscles. Averages of the different stunning methods were compared using ANOVA for mixed methods with “stunning method” as fixed and “slaughter day” as random effect. Where relevant, lactate or glycogen levels or pH were introduced as covariables into the ANOVA of certain meat quality indicators to evaluate whether effects of stunning method could be statistically explained by these quantitative explanatory variables. ANOVA for repeated analyses was used to compare muscles (repeated factor “muscle”; fixed effect “stunning method”).

## 3. Results and Discussion

All pigs were unconscious after stunning. A total of four pigs after immersion in N_2_O/CO_2_ and one after immersion in the CO_2_/air mixture showed leg movements at the end of bleeding, but no respiration. These movements are a sign of a risk of return of consciousness, which is obviously unacceptable, both in terms of animal welfare and security [25]. This observation is consistent with our earlier observation that pigs stunned with CO_2_/N_2_O stood sooner than when stunned with the CO_2_/air mixture [7]. This problem may be solved by longer dwelling durations or sooner bleeding than the 40 s delay practiced in the present study.

**Figure 2:**
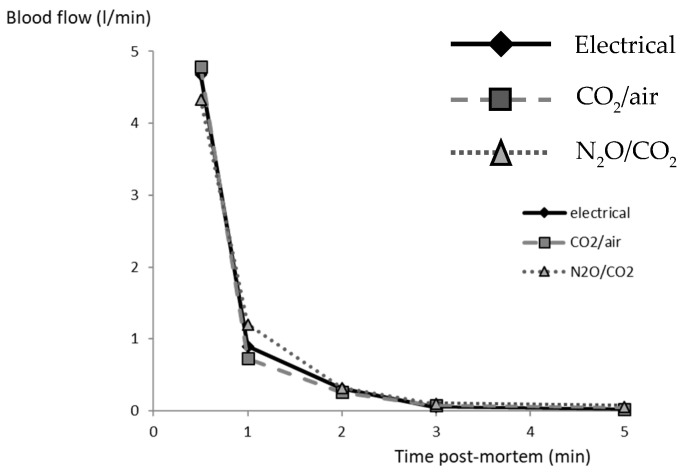
Blood flow (l/min) for five 60 s intervals following the thoracic bleeding cut, for the three stunning methods. Average SEM 0.39, 0.13, 0.06, 0.02 and 0.02 l/min for the respective time points.

Neither the quantities of blood collected over 5 min, nor the calculated flow rates, were influenced by the stunning method, with 2288 ± 230, 461 ± 80, 329 ± 80, 86 ± 30 and 103 ± 50 g for the successive intervals (total of 3163 ± 160 g; Figure 2). The total amount of blood collected correlated with carcass weight (r = 0.59, *p* < 0.001) and with the amount collected during the first 30 s (r = 0.69, *p* < 0.0001). Hence, the bleeding rates and total blood loss were similar and satisfactory for all pigs, irrespective of the stunning method. 

In total, 3 of the 10 electrically stunned pigs and a pig stunned with the CO_2_/air mixture had 3 or 4 blood spots on the SM slice (Chi square: not significant). The latter pig also had a fractured pelvis. The lack of significance is probably due to the reduced number of pigs used. However, the trend is consistent with literature data [15,16,17,18,20], which report higher incidences of petechiae after electrical than after CO_2_ stunning.

An electrically stunned pig and another pig stunned with the N_2_O/CO_2_ mixture had shown much physical resistance when introducing them in the metal cage for transport. Probably as a consequence of this, they had a low pH and low GP 5 min post-mortem. These pigs were excluded from the ANOVA analysis for meat quality aspects to avoid bias, but not for the correlations.

At 5 and 60 min, electrically stunned pigs had a higher LL pH than pigs stunned with the CO_2_/air mixture and at 5 min a higher LL pH than pigs stunned with the N_2_O/CO_2_ mixture (Table 2). LL lactate was lower (*p* < 0.01) 5 min after electrical stunning (22.0 ± 2.8 μmol/g) than after CO_2_/air (33.4 ± 1.7 μmol/g) or N_2_O/CO_2_ stunning (31.3 ± 2.2 μmol/g). Some other minor effects were observed. Thus, LL drip loss on day 5 was greater (*p* < 0.03) after CO_2_/air stunning (2.87 ± 0.17%) than after electrical (2.20 ± 0.21%) or N_2_O/CO_2_ stunning (2.32 ± 0.09%). Following N_2_O/CO_2_ stunning SM drip loss on days 1 (*p* = 0.08) and 2 (*p* < 0.05) was greater (total of 4.26 ± 0.37; *p* < 0.05) than following electrical (2.97 ± 0.16%) or CO_2_ stunning (3.42 ± 0.34%). *p*-values for the effects of stun method on GPs, and glycogen levels of the LL and SM were greater than 0.15. In summary, the effects of stunning method on LL and SM technological meat quality indicators concerned only a few indicators. Particularly, early post-mortem pH decline in the LL was faster following gas stunning than following electrical stunning, while the pH of other muscles were not influenced. Following CO_2_/air stunning, LL drip loss between days 2 and 5 post-mortem was higher, and following CO_2_/N_2_O stunning SM drip loss was higher the first two days, compared to the other methods, but overall drip loss over 6 days was not influenced by stunning method.

Finally, AF meat had lower lightness (*p* < 0.01) after CO_2_/air stunning (42.1 ± 0.8) compared to electrical (46.5 ± 1.0) or N_2_O/CO_2_ stunning (45.9 ± 0.7). No other effects of stunning method were found. Light meat color is generally associated with faster early post-mortem pH decline and/or lower ultimate pH [30], but electrical stunning had no effect on AF post-mortem pH decline. Possibly, the stunning method has influenced other mechanisms involved in meat color determinism [30].

**Figure 3:**
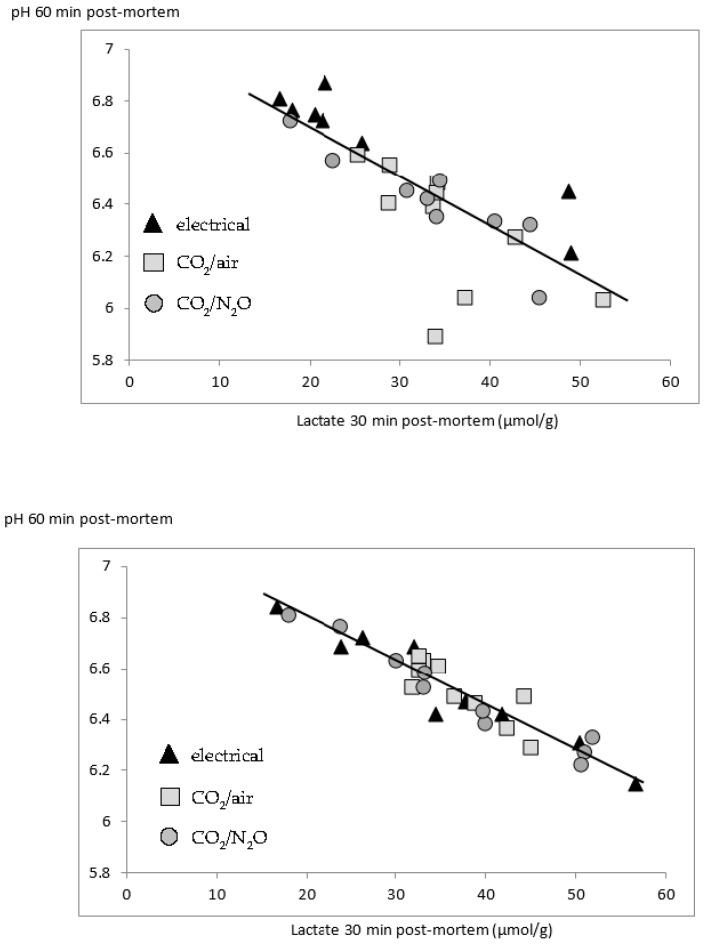
Correlation between the pH (60 min post-mortem) and lactate content (30 min post-mortem; µmol lactate equivalents per g of fresh tissue), for (**top**) *Longissimus lumborum* (r = −0.79; *p* < 0.001) and (**bottom**) *Semimembranosus* (r = −0.95; *p* < 0.001).

Correlation analysis on the overall LL data set found strong correlations between early post-mortem pH, and early glycogen and lactate contents, while correlations with temperature were weaker (Table 3; Figure 3). Ultimate pH was weakly correlated with GP, and more strongly with RN and DL (Table 3). RN was also positively correlated with muscle temperature 30 h post-mortem (Table 3). In a regression analysis on RN, both ultimate pH (*p* = 0.03), and temperature 30 h post-mortem (*p* = 0.02) were significant, but this effect was due to 3 pigs with relatively high RN values. 

Total DL was correlated with ultimate pH (r = −0.46; *p* = 0.01) and lactate 5 min post-mortem (r = 0.50; *p* < 0.01). In a regression analysis on total DL, ultimate pH was significant (*p* = 0.002), but not lactate content 5 min post-mortem (*p* = 0.35). Fitting ultimate pH as explanatory variable in the ANOVA on DL5 (ultimate pH: *p* < 0.0001) did not remove the effect of stunning method (*p* < 0.03). The introduction of the LL pH 5 min post-mortem in the analysis of variance cancelled the effect of the stunning on the lactate content measured 30 min post-mortem (pH effect: *p* < 0.0001, stun effect: *p* > 0.38). Glycogen content 5 min post-mortem was significant as quantitative variable (*p* < 0.05) but did not remove the significance of the effect of the stunning method on the pH 5 (*p* = 0.01) or 60 min post-mortem (*p* = 0.05). Glycogen content 30 min post-mortem was not significant (*p* > 0.22) as quantitative variable in the ANOVA of pH 5 or 60 min post-mortem. 

As for the LL, correlation analysis on the overall SM data set found correlations between early post-mortem pH, glycogen and lactate contents, but correlations with temperature were weak or absent (Table 3; Figure 3). Red index was weakly correlated with early post-mortem pH. Additional correlations for the SM were found between RN and lactate contents 30 h post-mortem (r = −0.49; *p* < 0.01); b* and L* (r = 0.37; *p* < 0.05); temperature 30 min post-mortem and glycogen content 30 h post-mortem (r = −0.37; *p* < 0.05); temperature 60 min and 30 h post-mortem (r = 0.52; *p* < 0.01) and DL2 and DL5. For the AF, early post-mortem pH was correlated with red index and temperature (Table 3). For the SC, the pH 30 min post-mortem was correlated with both the pH (r = 0.66; *p* < 0.001) and temperature 60 min post-mortem (r = −0.43; *p* < 0.05). Slaughter live weight was not correlated with any of the measured variables.

These correlations show that higher lactate contents early post-mortem explain, at least partly, the higher drip losses following gas stunning. This relationship is well known [31]. Post-mortem, muscle glycogen is degraded, producing energy, but also heat, hydrogen ions, and lactate. During the early post-mortem period, certain steps of the glycolysis produce hydrogen ions, while lactate production occurs once pyruvate has been formed [32]. Under the anaerobic conditions of the post-mortem muscle, the process cannot sustain the energy needs and progressively ATP levels lower. The glycolysis itself and the net hydrolysis of ATP result in the production of hydrogen ions leading to a pH decline [26,32,33]. The associations between higher muscle temperature, faster early post-mortem pH decline, higher lactate content and lower remaining glycogen reserves observed in the present study are coherent with a faster post-mortem metabolism. The fast pH decline while the muscle is still warm causes increased protein denaturation, which may explain the increased drip loss observed at certain time points following gas stunning [31]. The greater RN yield in meat with lower glycolytic potential and higher ultimate pH is coherent with existing knowledge [29]; none was influenced by stunning method. The effect on pH on meat color is also well known and related to its effects on myoglobin, among others [30].

Irrespective of stunning method, the SC had higher (*p* < 0.001) ultimate pH (5.68 ± 0.02) compared to the other muscles. AF had higher (*p* < 0.01) ultimate pH (5.45 ± 0.01) than LL (5.38 ± 0.01) and SM (5.39 ± 0.01). Muscles differed also in temperature at different times post-mortem (Table 4). Irrespective of stunning method, the AF muscle had lower lightness (*p* < 0.01), and higher yellow (*p* < 0.0001) and red index (*p* < 0.05) than the LL and SM muscles (Table 3). The higher pH of the SC and the AF and the different color of the AF muscle are at least partly explained by their different fiber composition [21,30].

The results obtained contrast with those earlier reported which were indicative of faster early post-mortem metabolism following electrical compared to gas stunning. Thus, in two field studies, Velarde et al. [15,16] found that abattoirs using electrical stunning produced lighter *Longissimus thoracis* (LT) meat with a higher risk of PSE compared to abattoirs using CO_2_ stunning, both indicative of a faster early post-mortem pH decline. Similarly, using an experimental abattoir to compare electrical and gas stunning, Channon et al. [17,18,20] found overall, faster early post-mortem pH decline after electrical stunning in the *Longissimus thoracis et lumborum* (LTL), and *Biceps femoris* (BF) muscles. Following electrical stunning, the LTL presented also greater drip loss [17,18,20]. Similar results were obtained by Marcon et al. [19].

The different results obtained in the earlier studies compared to the present study may be caused by higher and uncontrolled pre-slaughter stress levels in the former. Increased physical efforts of the animal just before slaughter accelerates ante-mortem muscle metabolism causing faster post-mortem glycogen breakdown and an increased rate of hydrogen ion, lactate and heat production [34,35,36]. Psychological stress due to fear of unfamiliar circumstances increases the secretion of adrenaline; this exacerbates the effects of physical activity on ante-mortem muscle metabolism and further accelerates early post-mortem energy metabolism [21,37,38,39]. The earlier studies by Velarde et al. [15,16], Marcon et al. [19] and Channon et al. [17,18,20] were conducted in commercial or pilot abattoirs. In these earlier studies, before stunning, pigs were more active and more psychologically stressed than in the present study, as the studies involved transport and driving of the walking animals and for the electrical stunning treatment, often the use of V-restrainers.

In the present study, pigs performed very little physical effort before slaughter, pre-slaughter psychological stress was low, as pigs did not walk but were wheeled to the stunning spot, and the delay between leaving the home pen and stunning was very short. During gas stunning, loss of consciousness is not immediate and during the induction, pigs are in respiratory distress and present uncontrolled muscle contractions [7]. Therefore, the faster pH decline and greater lactate production in the LL muscle in the gas compared to electrically stunned pigs probably results from the greater physical effort and/or psychological stress during the induction period, leading to a faster glycolytic rate. An earlier report suggests also that the involuntary muscle contractions during gas stunning may accelerate pH decline: when comparing different CO_2_/N_2_ mixtures, mixtures with lower CO_2_ and higher N_2_ concentrations were associated with increased involuntary muscular excitation, a faster pH decline and increased drip loss [40]. Other factors, such as gas concentration (a progressive increase in the earlier studies), the exact site of application of the stunning electrodes on the pig, or the genetic background of the animals were also different between the present and earlier studies [17,18] and may have contributed to their different results.

In both the LL and SM muscles, greater hydrogen ion content (lower pH) was strongly associated with greater lactate contents and greater lactate and hydrogen ion contents were associated with lower glycogen contents (faster glycogen breakdown). The latter correlations were weaker than the correlations between hydrogen ion and lactate contents, probably because animals differed in their initial muscle glycogen contents at the moment of slaughter. The correlation between the hydrogen ion and lactate production during the early post-mortem period explains up to 90% (based on the r^2^ for pH/lactate correlation) of the individual variability in the early post-mortem rate of pH decline in the SM. This suggests that glycolytic rate is the main cause of the effect of stunning method on pH decline: if ever the absorption of CO_2_ contributes to the acidification of the muscles because of its acidifying effects on blood, the effect is likely minor.

Like gas stunning, electrical stunning influences post-mortem metabolic rate, due to its effect on muscle contraction. Particularly, higher intensities, longer, or head-brisket (rather than head-only) current applications increased rate of pH decline and drip loss compared to controls [18,20], probably because of the increased contractions of the muscles. For a given muscle, its sensitivity to the electrical parameters may partly depend on their position on the body. For example, sites of the LTL, further removed from the vertebrae, were not influenced by the electrical parameters [17,18,20].

## 4. Conclusions

Both gas stunning and electrical stunning cause muscle contraction and consequently influence post-mortem muscle metabolism. When comparing electrical and gas stunning methods maintaining pre-stunning physical effort and psychological stress at minimal levels, differences in meat quality indicators are minor. Principally, gas stunning accelerated early post-mortem LL pH decline compared to electrical stunning. This faster early LL post-mortem pH decline was associated with greater drip loss between 3 and 6 days post-mortem, but overall drip loss was not influenced by stunning methods. Rate of pH decline was not influenced in the other muscles, indicating that they were affected similarly by electrical and gas stunning. Differences between electrical and gas stunning reported by earlier studies contrast with those reported here and are probably due to different levels of pre-slaughter stress and not to different impacts of the stunning method. This indicates that electrical stunning may result in improved meat quality if pre-stun conditions are less stressful and demand less physical effort. The addition of N_2_O to the gas mixture did not present any advantage with respect to meat quality indicators.

## Figures and Tables

**Table 1:** Main aspects of the protocol.

Treatments	N ^1^	Duration of Application
*Gas*: 80% of CO_2_ in air	10	90 s
*Gas*: 70% of N_2_O with 30% of CO_2_	10	90 s
*Electrical*: 230 V	10	10 s
**Measurements (all treatments)**		
Bleeding efficiency		
Carcass weight		
Muscle ^2^ pH decline and temperature:		
LL: 5, 30 and 60 min, and 30 h pm ^3^		
SM, AF and SC: 30 and 60 min, and 30 h pm		
Color (L*, a*, b*):		
LL, SM, SC: 30 h pm		
Water holding capacity:		
LL, SM: Drip Loss (DL) 1, 2 and 5 days pm		
LL: Rendement Napole (RN)		
Glycogen and lactate content:		
LL		

^1^. N = number of pigs used in the treatment. ^2^. LL = *Longissimus lumborum*; SM = *Semimembranosus*; AF = *Adductor femoris*; SC = *Semispinalis capitis*. ^3^. pm = *post-mortem*

**Table 2:** Mean pH values (±SEM) for the different muscles at different times post-mortem, according to stunning method.

Muscle	pH	Electrical	CO_2_/Air Mixture	N_2_O/CO_2_ Mixture
LL	5 min	6.67 ± 0.04 ^x^	6.52 ± 0.03 ^y^	6.52 ± 0.04 ^y^
	30 min	6.63 ± 0.07	6.49 ± 0.02	6.46 ± 0.06
	60 min	6.60 ± 0.09 ^a^	6.31 ± 0.08 ^b^	6.39 ± 0.06 ^ab^
	30 h	5.40 ± 0.03	5.37 ± 0.02	5.37 ± 0.01
SM	30 min	6.53 ± 0.07	6.50 ± 0.04	6.53 ± 0.07
	60 min	6.52 ± 0.08	6.51 ± 0.04	6.49 ± 0.07
	30 h	5.42 ± 0.01	5.38 ± 0.02	5.36 ± 0.03
AF	30 min	6.51 ± 0.06	6.45 ± 0.06	6.44 ± 0.07
	60 min	6.47 ± 0.05	6.39 ± 0.08	6.36 ± 0.09
	30 h	5.45 ± 0.02	5.45 ± 0.02	5.47 ± 0.03
SC	30 min	6.48 ± 0.04	6.51 ± 0.04	6.47 ± 0.03
	60 min	6.41 ± 0.04	6.46 ± 0.03	6.44 ± 0.05
	30 h	5.67 ± 0.05	5.69 ± 0.02	5.69 ± 0.02

LL = *Longissimus lumborum*; SM = *Semimembranosus*; AF = *Adductor femoris*; SC = *Semispinalis capitis*. Within a line, means with different superscripts are significantly different (x, y: *p* < 0.01; a, b: *p* < 0.05).

**Table 3:** Pearson correlation matrices for the LL, SM and AF muscles for variables at different times post-mortem, across stunning methods ^1^. Lines and columns without significant correlations have been removed. See text for additional correlations (SM, SC). *: *p* < 0.05; **: *p* < 0.01; ***: *p* < 0.001.

***Longissimus lumborum***												
	pH 5 min	pH 30 min	pH 60 min	pH 30 h	Glycogen 5 min	Glycogen 30 min	Glycogen 30 h	Lactate 5 min	Lactate 30 min	GP	RN	Drip loss day 2
pH 30 min	0.68 ***											
pH 60 min	0.69 ***	0.66 ***										
Glycogen 5 min	0.45 *	0.43 *	0.32	−0.23								
Glycogen 30 min	0.43 *	0.22	0.19	−0.34	0.64 ***							
Glycogen 30 h	−0.03	0.07	−0.10	−0.06	0.66 ***	0.45 *						
Lactate 5 min	−0.74 ***	−0.80 ***	−0.82 ***	−0.29	−0.37 *	−0.13	0.09					
Lactate 30 min	−0.78 ***	−0.69 ***	−0.79 ***	0.05	−0.34	−0.44 *	0.17	0.65 ***				
GP	0.14	0.10	−0.03	−0.38 *	0.90 ***	0.63 ***	0.79 ***	0.06	−0.07			
RN	−0.004	−0.03	0.12	0.47 **	−0.34	−0.12	−0.23	−0.16	−0.14	−0.44 *		
Temperature 5 min	−0.37 *	−0.33	−0.42 *	0.06	0.04	−0.11	0.13	0.24	0.42 *	0.15	−0.21	
Temperature 30 min	−0.14	−0.13	0.06	−0.03	−0.28	−0.44 *	−0.53 **	0.02	0.18	−0.29	0.06	
Temperature 30 h	0.17	−0.07	0.11	0.25	−0.15	0.09	0.00	−0.09	−0.20	−0.21	0.49 **	
Drip loss Day 3	−0.13	−0.37 *	−0.23	−0.34	−0.08	0.03	0.16	0.44 *	0.08	0.12	−0.44 *	0.44 *
Drip loss Day 6	−0.03	−0.11	−0.33	−0.60 ***	0.19	0.28	0.08	0.37 *	0.17	0.38 *	−0.47 **	0.20
***Semimembranosus***												
	pH 30 min	pH 60 min	Glycogen 30 min					Glycogen 30 h				
pH 60 min	0.94 ***											
Glycogen 30 min	0.66 ***	0.72 ***										
Glycogen 30 h	−0.02	0.05	0.55 **									
Lactate 30 min	−0.93 ***	−0.95 ***	−0.70 ***					0.01				
GP	0.28	0.35	0.88 ***					0.75 ***				
a*	−0.48 **	−0.40 *	−0.14					0.17				
***Adductor femoris***												
Variables	pH 30 min	pH 60 min	L *					a*				
pH 60 min	**0.83**											
a*	**−0.56**	**−0.50**	0.12									
b*	0.03	0.00	**0.50**					0.17				
Temperature 30 min	**−0.49**	**−0.46**	−0.31					**0.42**				

^1^. Significant correlations are in bold letter type.

**Table 4:** Mean values (± SEM) of variables measured for the different muscles at different times post-mortem (see Table 2 for pH values). Cells are empty when no measurements were made.

	Time Post-Mortem	LL	SM	AF	SC
Temperature	5 min	39.2 ± 0.1			
	30 min ^3^	40.2 ± 0.3 ^w^	37.3 ± 0.3 ^x^	38.3 ± 0.2 ^y^	36.8 ± 0.3 ^x,z^
	60 min ^3^	35.2 ± 0.4 ^w^	32.5 ± 0.5 ^x^	34.7 ± 0.4 ^w,y^	29.9 ± 0.6 ^z^
	30 h ^3,4^	4.5 ± 0.1 ^x^	4.7 ± 0.1 ^a,y^	4.7 ± 0.1 ^a,y^	4.6 ± 0.1 ^b,x,y^
Color	L* ^3^	54.8 ± 0.4 ^x^	54.1 ± 0.6 ^x^	44.8^2^ ± 0.6 ^y^	
	a* ^3^	7.2 ± 0.3 ^x^	7.6 ± 0.4 ^x^	13.6 ± 0.4 ^y^	
	b* ^3,4^	7.4 ± 0.3 ^a^	7.4 ± 0.4 ^b^	8.4 ± 0.4 ^b^	
Glycogen ^1^	5 min	45.9 ± 1.9			
	30 min	48.0 ± 1.9	42.5 ± 1.8		
	30 h	14.9 ± 1.2	13.2 ± 1.2		
Lactate ^1^	5 min	29.1 ^2^ ± 2.5			
	30 min	33.4 ± 0.7	36.4 ± 1.9		
	30 h	64.3 ± 3.4	66.9 ± 1.4		
GP ^1^	5 min	120.9 ± 3.4			
	30 min	126.3 ± 3.6	121.3 ± 2.7		
Drip Loss (% loss)	DL1	1.69 ± 0.2	1.95 ^2^ ± 0.2		
	DL2	1.52 ± 0.1	1.61 ^2^ ± 0.1		
	DL5	2.52 ^2^ ± 0.1	3.11 ± 0.1		
	Total	5.73 ± 0.3	6.66 ± 0.2		
	RN	9.60 ± 0.4	9.01 ± 0.4		

^1.^ Expressed in µmol lactate equivalents per g of fresh tissue; ^2.^ Effect of stunning method (see main text); ^3.^ Within a line, means with different superscripts w, x, y, z are significantly different (*p* < 0.005); ^4.^ Within a line, means with different superscripts a, b, are significantly different (*p* < 0.05).

## Data Availability

Data sharing is not applicable to this article.
